# New Mutation Associated with Polycystic Kidney Disease Type I: A Case Report

**DOI:** 10.3390/genes15101262

**Published:** 2024-09-27

**Authors:** Vanya Rai, Manisha Singh, Joseph H. Holthoff

**Affiliations:** 1 Mayo Clinic, 200 First Street SW, Rochester, MN 55905, USA; rai.vanya@mayo.edu; 2 Department of Nephrology, University of Arkansas for Medical Sciences, Little Rock, AR 72205, USA; jhholthoff@uams.edu

**Keywords:** case report, Congenital Anomalies of the Kidney and Urinary Tract, autosomal dominant polycystic kidney disease, Genetic Mutation, *PKD1*, CKD

## Abstract

Introduction: Autosomal dominant polycystic kidney disease (ADPKD) is one of the most prevalent heritable disorders, characterized by the progressive development of kidney cysts leading to renal failure. It is primarily caused by mutations in the *PKD1* and *PKD2* genes, which account for approximately 85% and 15% of cases, respectively. This case report describes a previously unreported mutation in the *PKD1* gene, identified in a family involving an aunt and her niece with ADPKD. Case Presentation: The index case, a 56-year-old female with chronic kidney disease stage 3b secondary to ADPKD and hypertension, exhibited a strong family history of polycystic kidney disease (PKD). Initial genetic evaluations did not identify any recognized pathogenic mutations, leading to a more detailed investigation which revealed a novel mutation in the *PKD1* gene. This mutation was also found in her niece, who presented with early-onset disease. Conclusions: The identification of a heterozygous six-nucleotide deletion, c.2084_2089del, resulting in the in-frame deletion of two amino acids, p.Pro695_Ala696del, in the *PKD1* gene, has been linked with ADPKD in these patients. This report emphasizes the need for continuous updates to genetic data for a deeper understanding of the diagnosis and prognosis of ADPKD that could potentially aid in targeted therapy.

## 1. Introduction

Autosomal dominant polycystic kidney disease (ADPKD) is a prevalent genetic disorder that leads to the progressive development of renal cysts, often resulting in chronic kidney disease (CKD) and end-stage renal disease (ESRD) [1]. The primary genetic culprits of ADPKD are mutations in the *PKD1* and *PKD2* genes, with *PKD1* mutations accounting for approximately 85% of cases and *PKD2* mutations responsible for about 15% [2]. The *PKD1* gene encodes polycystin-1, a protein integral to the maintenance of renal tubular architecture and function [3]. Polycystin-1 is a large protein composed of more than 4000 amino acids, and it plays a critical role in maintaining the architecture of renal tubular cells and regulating cellular signaling. The protein is known to interact with polycystin-2 in a complex that mediates calcium signaling, which is essential for normal kidney function. Disruptions in these genes lead to cyst formation, which compromises renal function over time [4]. Polycystin-1, along with polycystin-2 (encoded by *PKD2*), forms a complex that regulates intracellular calcium signaling and other critical cellular processes [5]. Disruptions in these genes lead to cyst formation, which compromises renal function over time. The clinical presentation of ADPKD is highly variable, influenced by the specific mutation and its impact on protein function, along with other genetic, epigenetic, and environmental factors [6].

Despite advancements in genetic screening, there are still cases where the causative mutation remains unidentified, underscoring the complexity of ADPKD and the need for continuous updates to genetic databases and screening techniques [7]. This case report highlights a novel mutation in the *PKD1* gene, expanding the mutation spectrum associated with ADPKD and emphasizing the importance of detailed genetic analysis in understanding and managing this disease.

## 2. Case Presentation

### 2.1. Patient A

Patient A is a 56-year-old female with a known history of hypertension, anemia of chronic disease, and chronic kidney disease (CKD) stage 3b, secondary to Autosomal Dominant Polycystic Kidney Disease (ADPKD). She has a significant family history of PKD, with multiple immediate and extended family members affected. Her father and all three of his brothers had ADPKD, while her mother developed end-stage renal disease (ESRD) before the age of 60, though the etiology remains uncertain. Both of Patient A’s sisters and her brother were diagnosed with ADPKD; her brother, who declined hemodialysis in his 40s, passed away subsequently. Additionally, her niece and nephew have also been diagnosed with cystic kidney disease. Notably, genetic testing did not reveal mutations in the common *PKD1* or *PKD2* genes.

Her hypertension is currently managed with a regimen of losartan, metoprolol, and amlodipine, with recent dosage adjustments due to persistent elevation of blood pressure. For anemia, treatment with ferrous sulfate was initiated due to iron deficiency. She has been evaluated for secondary hyperparathyroidism related to her renal condition, with findings of stable calcium and phosphorus levels but elevated parathyroid hormone (PTH). Patient A is now considering tolvaptan therapy, weighing its potential benefits and risks.

### 2.2. Patient B

Patient B is a 35-year-old female who was diagnosed with polycystic kidney disease around 10 years ago following the early onset of hypertension and reduced renal function, which is atypical for her age. Despite her condition, she currently denies any acute symptoms, such as nausea or hematuria, and maintains stable blood pressure readings at home. Her renal function, as indicated by serum creatinine levels, remains within normal limits, and she has no significant proteinuria. Her condition is managed with losartan combined with hydrochlorothiazide, along with lifestyle modifications, including a low sodium diet and increased fluid intake.

### 2.3. Clinical Findings

Upon initial evaluation, Patient A underwent renal ultrasound imaging, which confirmed extensive bilateral renal cysts, characteristic of ADPKD. The total kidney volume was significantly increased, correlating with her chronic kidney disease stage 3b. Her blood pressure was elevated and managed with antihypertensive medications. Laboratory tests highlighted a declining renal function with an estimated glomerular filtration rate (eGFR) of 35 mL/min/1.73 m^2^, indicative of moderate to severe renal impairment.

The niece, Patient B, presented with early signs of hypertension and reduced renal function, which is unusual given her relatively young age. Her renal ultrasound findings depicted multiple bilateral cysts distorting the renal parenchyma with no signs of hydronephrosis or perinephric fluid collection, and no renal calculi.

### 2.4. Genetic Results

Genetic testing was performed using the Natera Renasight panel, analyzing DNA extracted from buccal swabs of the patients. Next-generation sequencing focused on the *PKD1* gene, given its established association with ADPKD, leading to the identification of a novel mutation. Genetic testing in Patient A uncovered a heterozygous six-nucleotide deletion, c.2084_2089del, which translates to an in-frame deletion of two amino acids, p.Pro695_Ala696del, in the protein product. This mutation was previously uncharacterized in our dataset and external databases, like Broad GnomAD.

Subsequent analysis within our internal dataset revealed the same mutation in Patient A’s niece, supporting a likely pathogenic classification due to its segregation with the disease phenotype in the family. This finding, in conjunction with the known clinical progression of ADPKD in the family and the absence of the mutation in broader databases, suggests a strong correlation between the mutation and disease manifestation. Given these factors, along with the mutation’s genetic prediction to result in an in-frame deletion in the *PKD1* gene, the variant was classified as likely pathogenic.

## 3. Discussion

Autosomal Dominant Polycystic Kidney Disease (ADPKD) is a genetically heterogeneous disorder, and the variability in disease severity and progression observed in these cases suggests a complex interplay between genetic, environmental, and potentially epigenetic factors [3,6]. This study highlights the importance of continuous surveillance and documentation of new mutations in ADPKD. Although numerous studies have explored the genetic and phenotypic correlations in ADPKD, there remain aspects yet to be fully understood. Further research is essential to deepen our understanding of this relationship and to study disease progression. Such insights could improve our ability to predict patient prognosis and potentially lead to the development of new targeted therapies [8].

The identification of a novel six-nucleotide deletion in *PKD1* resulting in the in-frame deletion of two amino acids, p.Pro695_Ala696del, provides new insight into the genetic underpinnings of ADPKD. While it remains speculative, the proximity of this mutation to important structural domains and features in polycystin-1 suggests a potential disruption of its function at the molecular level. The mutation identified in this case occurs relatively early in the extracellular domain of the protein, around amino acids 695–696, a region that is known to contain several key functional domains.

Notably, UniProt database analysis shows that this mutation is situated near an atypical LDL-receptor class A domain. LDL-receptor class A domains are often involved in protein–protein interactions, and the proximity of this deletion to this domain suggests that it may impair the ability of polycystin-1 to interact with other molecules or proteins crucial for its function. Furthermore, the UniProt Feature Viewer also highlights the closeness of this mutation to several disulfide bonds, which are critical for the stability and proper folding of extracellular domains. The deletion of Pro695 and Ala696 could potentially disrupt these disulfide bonds, leading to misfolding or structural instability in this region of the protein.

Given the massive size of polycystin-1 and the complexity of its interactions, even small mutations like this two-amino-acid deletion could have considerable effects on protein function. While the exact functional impact of this deletion is yet to be determined, the mutation’s location in an extracellular region of the protein, near critical structural features, supports the hypothesis that it could contribute to disease progression by altering protein stability or function. Further functional studies would be necessary to assess the exact consequences of this mutation on the polycystin-1 protein and its role in ADPKD.

It is crucial to continuously detect and document new mutations while developing novel approaches to mutation detection to enhance the diagnostic and prognostic value of genetic testing for ADPKD [9]. ADPKD shows high phenotypic variability and a wide variation in the age of onset of ESRD [10]. Approximately 45% of patients reach ESRD by age 60 [11]. Notably, *PKD1* is associated with an earlier onset of ESRD—approximately 20 years earlier—compared to patients with *PKD2* mutations, highlighting a clear genotypic-phenotypic correlation in ADPKD [12]. The presence of more cysts and earlier progression in *PKD1*-related disease indicates the importance of distinguishing between *PKD1* and *PKD2* mutations, as the former is often associated with a more severe phenotype [13]. The identification of mutations in the *PKD1* or *PKD2* genes holds significant prognostic value, particularly in families with no known history of Autosomal Dominant Polycystic Kidney Disease (ADPKD). In cases where early-onset disease is present, detecting hypo-morphic alleles can be crucial for identifying siblings at risk and enabling interventions, such as preimplantation genetic diagnostics [14].

For over 20 years, the mutated gene has been recognized for its strong prognostic value. It was primarily correlated with the age at which patients reached ESRD [15]. However, the significance of allelic information is only now becoming apparent. A study highlighted a significant allelic effect in *PKD1*, showing that non-truncating mutations are associated with milder disease compared to truncating mutations [16]. Previous research demonstrated that the position of the *PKD1* gene mutation correlates with renal disease severity. Specifically, mutations located in the 5′ region of the gene were associated with more severe outcomes, with a median ESRD onset of 53 years compared to 56 years for mutations in the 3′ region [17]. However, the Genkyst cohort study by Cornec-Le Gall et al. (2013) later described a stronger effect of *PKD1* mutation type—rather than its position—on renal phenotype. The study highlighted that truncating *PKD1* mutations lead to an earlier onset of ESRD compared to non-truncating mutations, which tend to delay disease progression. Additionally, *PKD1* mutation carriers generally have a poorer renal prognosis and develop hypertension earlier than *PKD2* mutation carriers [7]. Another study has demonstrated that sex and specific allelic variations within the *PKD1* gene significantly influence the expression and severity of ADPKD. Male patients, for instance, often exhibit more severe manifestations, underscoring the modulatory role of sex on the genotype–phenotype relationship. The variability in disease progression is also attributed to the differential impact of distinct *PKD1* alleles, emphasizing the complexity of genetic contributions in ADPKD [18].

Cornec-Le Gall et al. developed the PROPKD Score, a model that combines clinical factors, such as the onset of hypertension, with genetic data, specifically *PKD1* and *PKD2* mutation types, to stratify patients into risk categories and provide a more accurate prognosis for renal survival [19]. Prognostic information in ADPKD can help identify patients with more severe disease who may benefit from more intensive clinical monitoring.

The discovery of this new mutation provides a significant addition to the catalog of known pathogenic mutations in ADPKD and shows the potential contribution of rare or private mutations to the disease burden in certain families [20]. The presence of this novel mutation in two affected family members, alongside the absence of recognized pathogenic mutations in common loci, supports the classification of c.2084_2089del as likely pathogenic. This classification is further reinforced by the critical functional role of polycystin-1 in maintaining renal tubular structure and function, and the predicted deleterious impact of the identified deletion on protein function [2,5,21].

Beyond the primary *PKD1* mutation, genetic analysis also revealed carrier mutations in NPSH2 and APOL1 in Patient B, genes associated with other kidney-related pathologies. While the *PKD1* and *PKD2* mutations are prevalent across all ethnic groups, there are some genetic variants more common in African American populations that may influence kidney disease progression. For example, the APOL1 gene variants, which are more common in African American populations, have been linked to an increased risk of chronic kidney disease (CKD) and could potentially modify the severity of ADPKD. In fact, APOL1 variants are known to contribute to faster progression to ESRD in African Americans, regardless of the underlying kidney disease [22]. These mutations were not directly implicated in the ADPKD phenotype observed in this family, which points towards the complexity of genetic interactions in renal diseases [23].

In the bigger picture, the identification of patients at risk for rapid disease progression is crucial not only for guiding treatment decisions but also for protecting patients who may not require aggressive therapy from the potential costs and side effects associated with treatment. Current clinical trials are investigating new therapeutic options, and careful patient selection based on genetic and clinical markers remains a key consideration in the development and implementation of these treatments [24]. There are currently 1178 known ClinVar of *PKD1* documented on the GnomAD browser, illustrating the genetic diversity and complexity of ADPKD.

## 4. Conclusions

The identification of the novel *PKD1* mutation, c.2084_2089del, broadens the mutation spectrum associated with ADPKD and emphasizes the need for detailed genetic investigations in atypical cases, both for personalized treatment approaches and for a deeper understanding of the disease at molecular level [25]. This case report shows the importance of considering rare genetic variants in patients with a strong clinical and familial history of ADPKD but without detectable mutations in commonly analyzed genes. This can help broaden our existing genetic knowledge of the disease and expand our understanding of the interplay of different mutations on the disease. With the current rapid advancement in the treatment of ADPKD, it is essential to identify the pathology of the disease to help us guide management. Basic genetic literacy is becoming increasingly important in clinical medicine, especially in diseases like ADPKD. We would need to research further to study the impact of this novel functional consequences of this mutation, its impact on phenotype of the disease, and to explore potential therapeutic interventions that may mitigate its impact on disease progression.

## Data Availability

The original contributions presented in the study are included in the article, further inquiries can be directed to the corresponding author.
